# Repeat Tracking of Individual Songbirds Reveals Consistent Migration Timing but Flexibility in Route

**DOI:** 10.1371/journal.pone.0040688

**Published:** 2012-07-25

**Authors:** Calandra Q. Stanley, Maggie MacPherson, Kevin C. Fraser, Emily A. McKinnon, Bridget J. M. Stutchbury

**Affiliations:** Department of Biology, York University, Toronto, Ontario, Canada; Liverpool John Moores University, United Kingdom

## Abstract

Tracking repeat migratory journeys of individual animals is required to assess phenotypic plasticity of individual migration behaviour in space and time. We used light-level geolocators to track the long-distance journeys of migratory songbirds (wood thrush, *Hylocichla mustelina*), and, for the first time, repeat journeys of individuals. We compare between- and within-individual variation in migration to examine flexibility of timing and route in spring and autumn. Date of departure from wintering sites in Central America, along with sex and age factors, explained most of the variation (71%) in arrival date at North American breeding sites. Spring migration showed high within-individual repeatability in timing, but not in route. In particular, spring departure dates of individuals were highly repeatable, with a mean difference between years of just 3 days. Autumn migration timing and routes were not repeatable. Our results provide novel evidence of low phenotypic plasticity in timing of spring migration, which may limit the ability of individuals to adjust migration schedules in response to climate change.

## Introduction

The degree to which long-distance migration is flexible in time and space is much debated [1]. Endogenous programs may control most of the variation in migration schedules [2], [3] or these may be largely flexible at the individual level in response to environmental conditions before departure and en route [4], [5]. Determining the degree of plasticity in migration schedules and routes is important for predicting responses to climate change [5]. Recent studies suggest that declines of long-distance migratory birds are a result of endogenous, relatively inflexible departure schedules from wintering sites in the tropics [6].

Inferences regarding the flexibility of migration schedules and routes have been largely restricted to observations at single breeding, winter, or stopover areas, providing only a snapshot of individual migratory behaviour. Observations of repeat migratory journeys of individual birds may yield important insights into the degree to which migratory programs are flexible, but data are rare due to the difficulty in continuous tracking of birds over large distances. Recent examples from birds large enough to carry satellite tags are illuminating; both osprey and marsh harriers showed relatively consistent migration timing, particularly in spring, but low route fidelity, suggesting strong endogenous control of schedules but relative flexibility to local conditions along migratory routes [7], [8]. New developments in direct-tracking technologies [9] now allow small birds to be tracked over an annual cycle [10], [11], [12], [13], [14], [15]. Using data from light-level geolocators, we compared between- and within-individual variation in migration timing and route of 45 individual songbirds (wood thrush *Hylocichla mustelina*) and examined repeat autumn and spring journeys of 10 individuals. If migration schedules are relatively fixed at the individual level, then departure date should strongly predict arrival date [2], and individuals should exhibit high repeatability in departure date from year to year [8]. We expected high repeatability in spring compared to fall migration schedules owing to stronger stabilizing selection on arrival date in temperate spring environments [16] and carry-over effects of breeding events on autumn migration schedules [12].

## Methods

### Ethics Statement

This study was conducted in accordance with the recommendations of the Ornithological Council ‘Guidelines to the Use of Wild Birds in Research’ and was approved by the York University Animal Care Committee (Animal Care Protocol Number: 2009-2 W (R1)). Governmental scientific permits for capture, handling and geolocator attachment were obtained in the U.S., Belize, and Costa Rica.

We used data from light-level geolocators (MK14S, 1.6 g, British Antarctic Survey) retrieved between 2008 and 2011 at a breeding site in Pennsylvania, USA (‘PA’, 41.8°N, 79.9°W, *n* = 30), and wintering sites in Costa Rica (‘CR’, 10.4°N, 84.0°W, *n* = 19) and Belize (‘BZ’, 16.6°N, 88.7°W, *n* = 7). Geolocators were attached to birds using a leg-loop harness [17] made of 2.5 mm Teflon ribbon [9]. Total weight of the geolocator and harness was 1.9 g or less which is ca. 4% of the weight (45.9 g ±4.3, n = 241) of wood thrush. Ten individual birds were tracked for at least 2 years (PA, *n* = 6; CR *n* = 4). Sex of each individual was determined by breeding characters or genetically; age class was determined by plumage characteristics. Our total data set consisted of 56 fall and spring migration tracks, including 9 individuals (1 female, 8 males) tracked twice and one individual tracked three times (female). Eleven birds (7 CR and 4 BZ) tagged at wintering sites were tracked on their first spring migration, but none of these were tracked in subsequent years. Mean breeding latitude of all birds was 41.5°N (range 33.2–46.9°N); 82% of birds bred within ±2.5° of the mean.

#### Geolocator analyses

Light data were analyzed using BASTrak software package (British Antarctic Survey). Raw light data were adjusted for any clock drift (typically <3 min.). Sunrise and sunset were defined as light transitions where the light levels crossed a threshold of 16 (2008 model) or 5 (2009–2010 model). These thresholds represent similar light intensities, based on static calibration of geolocators in known locations. Light transitions were then visually inspected and edited using the program TransEdit to delete false sunrises and sunsets (e.g. transitions during daytime caused by shading) and to score the quality of true sunrise and sunset transitions. The slope of the light data at dawn or dusk was visually compared to transition slopes from static geolocators with a full sun exposure. Very shallow slopes were marked as low confidence, as were transitions that included small peaks in light intensity prior to reaching sunrise threshold, or after reaching sunset threshold. In these cases, the marked transition was unlikely to be within 10 minutes of the actual sunrise/sunset transition and so was excluded from subsequent analysis. Only the transitions with a high confidence score were used in further analyses. After each light data file was edited, we used the program Locator (BAS) to transform light data into latitudinal and longitudinal positions and used a sun elevation angle calculated using season-specific data [18] gathered from birds carrying geolocators at known breeding and wintering sites.

#### Movement analyses

We relied primarily on longitude to determine timing of movements, since error in longitude is much smaller than error in latitude and longitude is not affected by the equinoxes, whereas latitude cannot be determined near the equinox (day length is the same everywhere). Position estimates may be influenced by topography, weather, seasonal changes in behaviour, and vegetation structure [19]. The influence of these factors on longitudinal position error is expected to be low as compared to latitude, but has not been quantified. Our study is unique, in that we deployed geolocators at both temperate breeding and tropical wintering locations enabling ground-truthing of position estimates. We used data retrieved from birds carrying geolocators at a winter site in Costa Rica (n = 15) and at a Pennsylvania breeding site (n = 23 birds) to calculate sun elevation angles for determining unknown breeding and winter sites. Using season- and location-specific sun elevations resulted in average error in longitude of 55±18 km (mean ±95% CI) at tropical winter sites, and 105±29 km at temperate breeding sites. In a temperate, non-migratory thrush, longitude error using geolocators was 50±34 km [18]. It is impossible to ground-truth position estimates during migration, but we assume similar error in longitudinal position during migration.

Movements away from breeding or wintering sites were defined as shifts in longitude greater than 2° in a direction consistent with migration; such shifts were typically accompanied by strong shifts in latitude consistent with migration direction. Arrival dates at breeding and wintering sites were determined when longitudinal values no longer shifted in a direction consistent with migration, varied less than 2°, and remained similar throughout the breeding or wintering period. Autumn departure date was unobtainable for many birds because migration was due south (i.e. primarily shifting in latitude) and thus position was masked by the autumnal equinox. Therefore, we used the date birds crossed 23.4°N (entry to Tropics) as a measure of timing of migration as it occurred well after the equinox period [12]. We calculated autumn migration distance for the final leg of the trip, between crossing of 23.4°N and wintering sites. To test for spatial repeatability of migration routes, we used longitude crossing 23.4°N (Tropic of Cancer) in both spring and autumn. The Tropic of Cancer coincides with a large migration barrier for wood thrushes, the Gulf of Mexico, and therefore is the most biologically important point on the route. Preliminary examination of our migration data suggested that birds could cross this barrier by several routes which also had a strong effect on subsequent final route to the breeding site. Route repeatability of satellite-tracked harriers was estimated at three latitudes along migratory routes [8]. Geolocator error in latitude is about twice that for longitude, and latitude cannot be determined within two weeks of the autumn and spring equinox. Thus, to retain the highest accuracy in route assignment, we choose a latitude that represents a migration barrier (Gulf of Mexico; 23.4°N) where stopovers are not possible, thus timing of crossing is usually discernible, and where the range of possible routes was maximized (i.e. routes differ by at least 100 km in longitude). Measuring repeatability at a more southerly latitude, within the tropics and prior to crossing the Gulf, would not be informative because wood thrush are naturally funneled by a narrow land mass (Yucatan Peninsula) as they pass between the Gulf of Mexico and wintering sites.

We examined three migration variables (date and longitude at cross of 23.4°N and arrival date) that are directly comparable between autumn and spring migration. The autumn equinox made it impossible to obtain departure dates for birds that did not substantially shift longitude on departure. Migration pace and duration is therefore not directly comparable between seasons. In autumn we measured pace and duration beginning at 23.4°N (i.e. the last leg of the trip) whereas in spring the pace and duration reflected the entire journey. However, timing of crossing 23.4°N in autumn is influenced by events at breeding sites [12] and in the subset of birds for which data were available, departure date was significantly correlated with date of crossing into tropics (F_1,19_ = 7.65, R^2^ = 0.25, p<0.01).

#### Variation in migration timing and repeatability analyses

To explore factors influencing variation in spring and winter arrival date of all birds (*n* = 56), we fit general linear models with departure date, breeding latitude, sex, and age (spring only, 1^st^ spring migration or not) as factors. We used tools in R that use a backwards step procedure (“step” function, R Development Core Team 2011) to drop individual explanatory variables one by one, refit the model each time, and then used Akaike’s Information Criterion (AIC) to measure model fit and complexity and select the optimal model [20]. With individual as a factor in an ANOVA, we compared between- and within-individual variation in migration timing and route [8]. We also determined the repeatability of time and space factors of 10 birds tracked in more than one year [21]. The same temporal and spatial variables (Table 1) were used in both analyses. Some migration variables were not available for all birds due to equinox, missing days, and battery failure. All analyses were conducted using R (R Development Core Team 2011).

**Table 1:** *p*-values of one-way ANOVA testing the effects of individual on migration variables of wood thrushes.

Variable	df	f	*p*-value
**autumn migration**			
date cross 23.4°N	38,8	3.13	0.77
longitude crossing 23.4°N	39,8	1.63	0.28
winter arrival date	43,8	1.60	0.25
Duration	42,8	1.58	0.25
**spring migration**			
departure date	42,9	4.27	0.01*
date cross 23.4°N	44,10	3.13	0.03*
longitude crossing 23.4°N	43,10	0.70	0.80
breeding arrival	43,9	4.69	0.009**
duration	51,8	2.74	0.07

Total of 56 individual fall and spring migrations tracked, including 9 individuals tracked twice and one individual tracked three times. Significance level indicated by asterisks: *p<0.05; **p<0.01, ***p<0.001.

## Results

**Figure 1:**
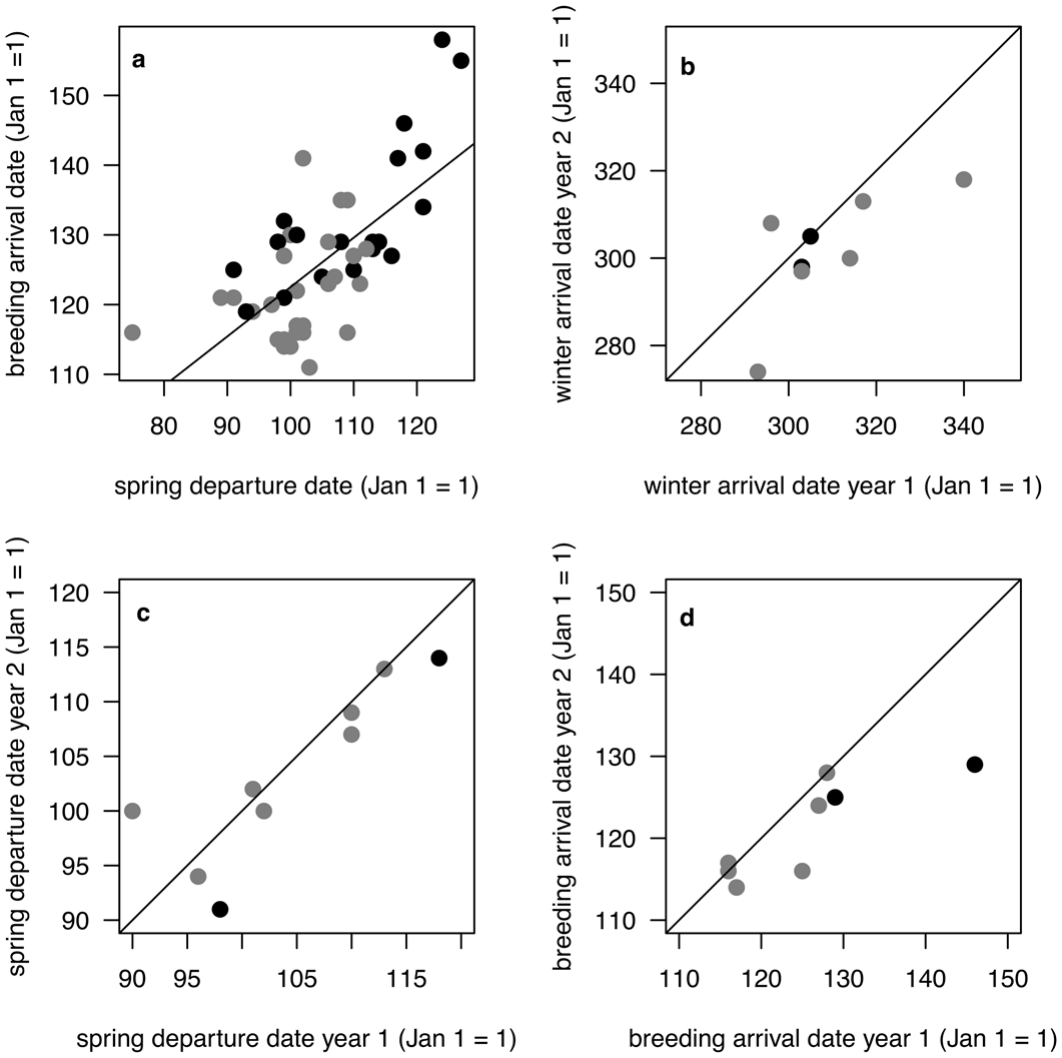
Migration schedules of wood thrush. (a) Spring departure date versus breeding arrival date of 56 migration tracks of 45 different individual wood thrushes (line shows least-squares regression). Black data points indicate female, grey male. Migration timing for individuals tracked in two consecutive years: (b) winter arrival date, (c) spring departure date, (d) breeding arrival date. For b–d, lines show 1∶1 relationship.

The timing of spring departure from Central America explained much of the variation (71%) in arrival dates at breeding sites, along with sex and age factors (F_4,45_ = 31.47, R^2^ = 0.71, p<0.001) (figure 1a). Spring departure and breeding arrival were positively correlated (model estimate 0.42±0.09 SE, t = 4.5, p<0.0001). As expected, males arrived earlier than females (estimate of −8.09±1.74 SE days, t  =  −4.56, p<0.0001) and birds on their first spring migration arrived later than birds that had migrated at least once before being tracked (estimate 12.48±2.24 SE days, t = 5.57, p<0.0001).

Autumn date crossing 23.4°N, was the only factor retained in the minimum adequate model but explained only 25% of the variation in winter arrival date (F_1,45_ = 16.34, R^2^ = 0.25, p<0.001). Individual had a significant effect in all spring migration timing variables (spring departure date, date crossing 23.4°N, breeding arrival date) except for spring migration duration (Table 1 and electronic supplementary material, Table S1). There was no effect of individual on autumn migration timing, or longitude at 23.4°N in spring or autumn (Table 1). Within-individual repeatability tests showed similar results: spring migration timing was more repeatable than longitude at 23.4°N, and spring variables were more repeatable than autumn variables (Table 2). The timing of winter arrival, spring departure, and breeding arrival were highly repeatable (*r*- values 0.62–0.71) (figure 1b, c, d) and spring departure dates (r = 0.71) differed between years by an average of ±3 d. Longitudes of migratory routes had low repeatability in both spring and autumn.

**Table 2:** Repeatability (*r*) of migration timing and route variables for wood thrushes tracked in two consecutive years (*p<0.05; **p<0.01, ***p<0.001).

variable	df	f	*r*	*p*-values
**autumn migration**				
date cross 23.4°N	7, 8	0.44	0.05	0.44
longitude crossing 23.4°N	9,10	1.28	0.12	0.43
winter arrival date	8, 9	4.20	0.62	0.02*
autumn migration duration	6,7	2.82	0.48	0.10
**spring migration**				
spring departure date	9, 10	5.94	0.71	0.005**
date cross 23.4°N	9, 10	2.90	0.49	0.07
longitude crossing 23.4°N	9, 10	1.28	0.12	0.35
breeding arrival date	8, 9	4.96	0.66	0.01*
spring migration duration	8, 9	2.37	0.41	0.11

## Discussion

Our results, based on comparisons among and within individuals, suggest that the timing of songbird migration in spring is under strong endogenous control and highly repeatable from year to year. For all birds, spring departure date was a significant predictor of breeding arrival date; differing departure dates and spring routes did not uncouple the relationship between departure and arrival. Considering the 27-day range of departure dates from wintering sites, and that comparisons were made over multiple years and presumably variable environmental conditions, it is surprising that departure dates of individuals tracked in multiple years were highly consistent between years. The high repeatability in spring departure date suggests a stronger influence of endogenous schedules than local environmental conditions, in contrast to a recent study of a Neotropical migrant warbler with strong social dominance that influences individual access to food, where departure dates were only 38% repeatable [4]. Spring migration in wood thrush is thus more similar to that of a long-distance migratory shorebird (bar-tailed godwit, *Limosa lapponica baueri*) where spring departure date from wintering sites in New Zealand was a strong predictor of arrival at Alaskan breeding sites [2] and spring departure date was highly repeatable between years [22]. However, the stronger relationship between spring departure date and breeding arrival date in wood thrush, than in godwit [2], may be explained by flexibility in the latter species in response to favourable wind conditions that mediate initial spring departure date [23].

Timing was less repeatable at date of crossing 23.4°N, which implies flexibility in migration timing en route to breeding sites [5], particularly around the period when birds cross a major open-water migration barrier, the Gulf of Mexico. These results are compatible with recent ringing studies suggesting some en route flexibility of songbird migration pace [24], [25] likely in response to weather and quality of stopover sites. In wood thrushes, breeding arrival dates, although repeatable, were on average 4 days earlier in the second year of tracking than the first (6 of 8 birds came back earlier in year 2). Earlier arrival at breeding sites by older individuals is well established by mark-recapture studies and is driven by sexual selection [26]. Age and experience likely improve the fine-tuning of migration schedules and [27] reduce the fitness costs of coping with inclement early weather during early spring. Direct-tracking studies comparing repeat migratory journeys of adult and juvenile songbirds have not been performed but would be invaluable for understanding the mechanisms driving differences in migration schedules between age classes.

Winter arrival date was also consistent for individuals and en route timing (date crossing 23.4°N) explained 25% of the variation in arrival date. Field and laboratory studies indicate that autumn departure date is heritable in songbirds and largely under endogenous control [3], [16]. In wood thrushes, timing of crossing 23.4°N in autumn was correlated with departure date from breeding sites but is also dependent on individual timing of molt and physiological condition prior to migration [12]. Timing and pace of autumn migration is flexible because late-breeding birds tended to moult and migrate later, though did not arrive later at the wintering territory owing to long stopovers by many birds en route [12], [28]. Migration timing was generally more repeatable in spring than fall migration. While it would be ideal to compare repeatability of spring versus autumn migration over the entire journey, due to overlap of migration with the fall equinox we could only examine autumn migration during the latter portion of the trip.

In contrast to migration timing, migration route (as measured by longitude after crossing the 23.4°N), had relatively low repeatability in spring and autumn. This suggests that route is not under strong endogenous control, and may be influenced by individual energetic condition and weather patterns. A similar flexibility in migratory routes, but not timing, was found in migratory raptors [7]–[8]. As with harriers [8], the variation we observed in longitude may reflect a fine-tuning of migration in response to local conditions, within the constraints of timing cued by winter photoperiod and selection for optimal arrival at breeding areas.

**Figure 2:**
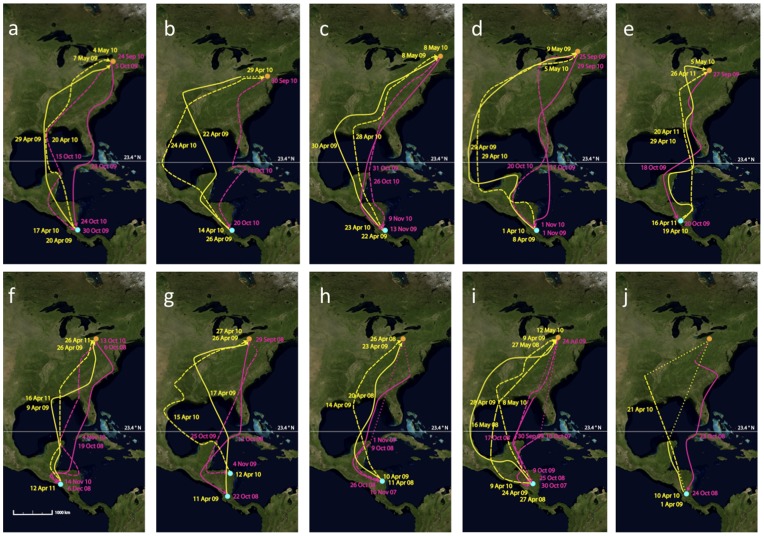
Repeat migration routes of individuals tracked in consecutive years by geolocator deployment. Birds were tracked from (a) Pennsylvania (b) Costa Rica. Yellow  =  spring migration, pink, pink  =  fall migration. Orange circle  =  breeding site, blue  =  winter site. Short-dashed lines indicate migration tracks in the second year and long-dashed lines a third year. Dotted lines indicate where migration route was unknown due to poor-quality light data, or geolocator battery failure.

Our low repeatability estimates for routes compared with timing of migration may occur in part if there are large differences in measurement error between these two aspects of migration behaviour. The precision of light-level based geolocation data in estimating location, even for longitude, is likely low compared with timing of major migration movements. We quantified spatial error in longitude (55±18 km, mean ±95% CI) based on data obtained from wood thrushes carrying geolocators at known wintering sites (McKinnon et al. in prep.), although error estimates during migration may be higher because there are fewer days on which to base locations. Migration timing was also based largely on longitudinal shifts, defined using the same longitudinal error estimates from ground-truthing. Since both spatial and temporal measures of migration depend on longitude, error may be comparable. Unfortunately, it is not possible to ground truth timing of migration and estimate error since a bird’s movements can only be determined from the geolocators themselves. However, within­individual route differences in longitude from year to year typically deviated by more than several hundred km (see Fig. 2 g), which is greater than the measurement uncertainty. Start-to-finish spring route of many individuals were substantially different between years (Fig. 2) which contrasts dramatically with the low within-individual variation in timing of spring departure (Fig. 1).

Repeatability is a measure of individual consistency relative to other individuals in the population. Spring departure differed by only 3 days, on average, for individuals from one year to the next which is surprising considering that departure dates in the population spanned 30 days. In contrast, longitude of spring migration route at 23.4°N was highly flexible for some individuals (8–10° difference between years) and nearly spanned the population-level range in spring route (12° longitude). Within-individual differences in spring or fall route (Fig. 2) may reflect flexibility to inter-annual variation in local environmental conditions en route such as wind, availability of suitable stopover habitat and potential interactions with variation in the physical condition of the bird itself [26], [29]. Low route fidelity suggests that birds may employ a complex interaction of compass mechanisms [30] to navigate to goal areas, such as breeding or wintering sites, using different routes [7]. More birds crossed the Gulf of Mexico in spring than in autumn, which may reflect a time-minimization strategy, consistent with selection for early breeding arrival, or may be driven by seasonal variation in wind conditions and fueling opportunities [10]. Investigation of temporal variation in environmental factors at key stopover sites and barriers (Gulf of Mexico) can allow tests of hypotheses for the remarkable within-individual and inter-seasonal differences in route we observed in wood thrushes.

Overall, our results show that migration schedules are more consistent among individuals and more repeatable within individuals than migratory routes, particularly in spring. Consistent schedules, based on tracking of individual osprey [7], godwits [2], harriers [8], and songbirds (this study) may reflect strong stabilizing selection on the timing of migration in long-distance migrants [16]. We found stronger repeatability, and coupling of departure and arrival dates, for spring migration than autumn migration. Strong endogenous control of spring migration is expected because early arrival may increase mortality during cold periods [31] and late arrival reduces reproductive success [16], [32]. Inflexible response of migration schedules to climate change has been implicated in population declines of long-distance migratory birds [33]. Understanding how inflexible spring migration schedules affect fitness of forest songbirds like the wood thrush is important for interpreting population declines.

## Supporting Information

Table S1 **Results of ANOVA testing the effects of individual on migration variables of wood thrushes.** Total of 56 individual fall and spring migrations tracked, including 9 individuals tracked twice and one individual tracked three times. Significance indicated in brackets (n.s., *p<0.05; **p<0.01, ***p<0.001).
(DOC)
