# Supplementary material for: Repeat Tracking of Individual Songbirds Reveals Consistent Migration Timing but Flexibility in Route
Source: PLoS One. 2012 Jul 25;7(7):e40688. doi: 10.1371/journal.pone.0040688 (PMC3405083; doi:10.1371/journal.pone.0040688)
Supplement: Table S1 — Results of ANOVA testing the effects of individual on migration variables of wood thrushes. Total of 56 individual fall and spring migrations tracked, including 9 individuals tracked twice and one individual tracked three times. Significance indicated in brackets (n.s., *p<0.05; **p<0.01, ***p<0.001). (DOC) [file pone.0040688.s001.doc]

*Electronic Supplementary Material (ESM)*

**Table S1.** Results of ANOVA testing the effects of individual on migration variables of wood thrushes. Total of 56 individual fall and spring migrations tracked, including 9 individuals tracked twice and one individual tracked three times. Significance indicated in brackets (n.s., **p*<0.05; ***p*< 0.01, ****p*< 0.001).

| **migration variable** | **factor** | **n** | **df** | **ms** | **f** | **p** |
| --- | --- | --- | --- | --- | --- | --- |
| **autumn migration** |  |  |  |  |  |  |
| date cross 23.4°N (autumn) | individual | 47 | 38 | 60.39 | 0.72 | 0.77 (n.s.) |
|  | residuals |  | 8 | 83.77 |  |  |
| longitude at autumn cross 23.4°N | individual | 48 | 39 | 11.329 | 1.63 | 0.28 (n.s.) |
|  | residuals |  | 8 | 6.936 |  |  |
| winter arrival date | individual | 52 | 43 | 131.26 | 1.60 | 0.25 (n.s.) |
|  | residuals |  | 8 | 81.83 |  |  |
| autumn migration duration | individual | 51 | 42 | 133.32 | 1.58 | 0.25 (n.s.) |
|  | residuals |  | 8 | 84.52 |  |  |
| **spring migration** |  |  |  |  |  |  |
| spring departure date | individual | 52 | 42 | 110.84 | 4.27 | 0.01 (*) |
|  | residuals |  | 9 | 25.96 |  |  |
| date cross 23.4°N (spring) | individual | 52 | 44 | 105.29 | 3.13 | 0.03 (*) |
|  | residuals |  | 10 | 33.62 |  |  |
| longitude at spring cross 23.4°N | individual | 54 | 43 | 8.273 | 0.70 | 0.80 (n.s.) |
|  | residuals |  | 10 | 11.821 |  |  |
| breeding arrival date | individual | 53 | 43 | 116.01 | 4.69 | 0.009 (**) |
|  | residuals |  | 9 | 24.74 |  |  |
| spring duration | individual | 50 | 51 | 72.44 | 2.74 | 0.07 (n.s.) |
|  | residuals |  | 8 | 26.46 |  |  |
